# 53BP1 promotes microhomology-mediated end-joining in G1-phase cells

**DOI:** 10.1093/nar/gku1406

**Published:** 2015-01-13

**Authors:** Xiahui Xiong, Zhanwen Du, Ying Wang, Zhihui Feng, Pan Fan, Chunhong Yan, Henning Willers, Junran Zhang

**Affiliations:** 1 Department of Radiation Oncology, School of Medicine, Case Western Reserve University, 10900 Euclid Avenue, BRB 323, Cleveland, OH 44106, USA; 2 Department of Oncology, Sidney Kimmel Comprehensive Cancer Center, Johns Hopkins University School of Medicine,1650 Orleans Street, Baltimore, MD 21231, USA; 3 Department of Biochemistry and Molecular Biology, Georgia Regents University, 1410 Laney Walker Blvd., CN-2134, Augusta, GA 30912, USA; 4 Department of Radiation Oncology, Massachusetts General Hospital, 55 Fruit Street, Boston, MA 02114, USA

## Abstract

Alternative non-homologous end joining (alt-NHEJ) was originally identified as a backup repair mechanism in the absence of classical NHEJ (c-NHEJ) factors but recent studies have demonstrated that alt-NHEJ is active even when c-NHEJ as well as homologous recombination is available. The functions of 53BP1 in NHEJ processes are not well understood. Here, we report that 53BP1 promotes DNA double-strand break (DSB) repair and genomic stability not only in c-NHEJ-proficient but also -deficient human G1-phase cells. Using an array of repair substrates we show that these effects of 53BP1 are correlated with a promotion of microhomology-mediated end-joining (MMEJ), a subtype of alt-NHEJ, in G1-phase. Consistent with a specific role in MMEJ we confirm that 53BP1 status does not affect c-NHEJ. 53BP1 supports sequence deletion during MMEJ consistent with a putative role in facilitating end-resection. Interestingly, promotion of MMEJ by 53BP1 in G1-phase cells is only observed in the presence of functional BRCA1. Depletion of both 53BP1 and BRCA1 increases repair needing microhomology usage and augments loss of DNA sequence, suggesting that MMEJ is a highly regulated DSB repair process. Together, these findings significantly expand our understanding of the cell-cycle-dependent roles of 53BP1 in DSB repair.

## INTRODUCTION

DNA double-strand breaks (DSBs) may cause cell death or genomic instability if not properly repaired (1,2). Homologous recombination (HR) and classical non-homologous end-joining (c-NHEJ) are the two major pathways for the repair of DSBs. c-NHEJ, which involves direct ligation of the broken DNA ends with or without limited end-processing, is the main mechanism for DSB repair in the G1-phase of the cell cycle though it can occur in other cell cycle phases as well (3–5). c-NHEJ is mediated by the DNA–PK complex, composed of a heterodimer of the Ku proteins, Ku70 and Ku80, and a catalytic sub-unit, DNA–PKcs. Religation of ends is achieved by the XRCC4–ligase IV complex (6). The endonuclease Artemis may be involved in processing of ends prior to religation, particularly if they contain complex DNA damage (7,8).

Alternative NHEJ (alt-NHEJ) was initially identified as a backup mechanism to repair DSBs when c-NHEJ is compromised (9,10). However, recent studies indicate that alt-NHEJ occurs even in cells proficient for c-NHEJ (11–13). Similar to c-NHEJ deficiency, alt-NHEJ defects confer radiation sensitivity (14,15). XRCC1, DNA ligase III and PARP-1 have central roles in alt-NHEJ (16–24). Alt-NHEJ is suppressed by Ku but promoted by CtIP (18,25,26). Alt-NHEJ typically requires short patches of perfectly matched sequences known as microhomologies (27,28). This type of rejoining is commonly referred to as microhomology-mediated end-joining (MMEJ) although not all alt-NHEJ events require microhomology. Alt-NHEJ is associated with the generation of 3′ single-strand overhangs which involves the MRE11/RAD50/NBS1 (MRN) complex and CtIP (8,16,18,29–32). This repair process typically relies on more extensive processing and sequence deletion than seen with c-NHEJ though the mechanisms and factors involved remain largely unknown.

53BP1 is involved in the DNA damage response but has no or a very limited role in activating cell-cycle checkpoints (33–36). 53BP1-deficient mice are growth-retarded, immunodeficient, predisposed to cancer and hypersensitive to radiation (33). The dramatic radiation sensitivity suggests that 53BP1 has a role in NHEJ processes but data have been conflicting (33–35). No obvious function of 53BP1 in c-NHEJ has been detected (33,35,37). Yet, 53BP1 promotes re-joining of distal DNA ends during V(D)J recombination, class switch recombination and the fusion of deprotected telomeres (38–40). In addition, 53BP1 may be involved in heterochromatin-associated DSB repair in G0/G1 phase cells (38,39,41). Importantly, 53BP1^−/−^ DT40 cells are radiation sensitive in G1-phase but the underlying mechanisms are unknown (34,35). These findings prompted us to investigate whether 53BP1 is involved in alt-NHEJ, and specifically MMEJ, in G1-phase cells.

53BP1 is frequently lost in triple-negative breast cancers, particularly those with BRCA mutation (42). 53BP1 inhibits HR and protects DNA ends from resection in BRCA1-deficient cells (42,43). 53BP1 and BRCA1 occupy associated yet mutually exclusive chromatin subcompartments at sites of DSBs, with 53BP1 exclusion from such sites occurring in a BRCA1- dependent manner during S phase (44). 53BP1 was found to promote HR via facilitating ssDNA resection in G2 phase cells, whereas it has no obvious role in HR in S phase cells (45). BRCA1 and 53BP1 oppose each other as further demonstrated by the observation that deletion of 53BP1 reduces mammary tumorigenesis and rescues PARP inhibitor sensitivity and viability of BRCA1-deficient mice (43,46). Despite extensive studies on the crosstalk of 53BP1 and BRCA1 in HR and DNA resection, the role of 53BP1 in NHEJ as a function of BRCA1 status has not been addressed.

Here, we have discovered a novel role of 53BP1 in promoting genomic stability and deletion-associated MMEJ in G1-phase cells. These functions are independent of DNA-PK but are modulated by BRCA1. Thus, our studies significantly expand our understanding of the role of 53BP1 in DSB repair across different phases of the cell cycle.

## MATERIALS AND METHODS

### Cell lines, infections and transfections

H1299 and U2OS cells were cultured in Dulbecco's modified Eagle medium (DMEM, Invitrogen) supplemented with 10% bovine calf serum, 100 U/ml penicillin and 100 μg/ml streptomycin at 37°C, 5% CO_2_. M059J cells were grown in a 1:1 mixture of DMEM and Ham's F-12 medium (DMEM/F-12, Gibco and Life Technologies), with 2.5mM L-glutamine adjusted to contain 15 mM HEPES, 0.5 mM sodium pyruvate and 1.2 g/l sodium bicarbonate supplemented with 0.05 mM non-essential amino acids and 10% fetal bovine serum (FBS). BRCA1 short-hairpin RNA (shRNA) has been described previously (47). The shRNAs targeting 53BP1, Ku70, Mre11, XRCC1, DNA ligase III, DNA-PKcs and CtIP were purchased from Sigma. All DNA plasmid transfections were performed using Lipofectamine 2000 according to the manufacturer's recommendations (Invitrogen, Carlsbad, CA, USA). H1299 cells with chromosomal integration of the MMEJ reporter were generated according to a standard protocol (48). In brief, H1299 cells were transfected with linearized pCMV/cyto/myc/GFP* substrate (also known as pCAM-1810) (30) or pPHW1. Stable integrants were selected and single-copy integration was validated by Southern blots using standard techniques. The sequences of primers for probe of H1299-pCAM-1810 are Primer-F: 5′-TCCCACTGTCCTTTCCTAATAAAAT-3′, Primer-R: 5′-CTCCTCACTACTTCTGGAATAGCTC-3′. The sequences of primers for probe of pPHW1 are Primer-F: 5′-GTGTCAGTTAGGGTGTGGAAAGT-3′, Primer-R: 5′-GGGATATCAACAACATAGTCATCAA-3′. The adenoviral I-SceI endonuclease expression vector, Ad-SceI-NG, was obtained from Kristoffer Valerie (49).

### MMEJ assays with pCMV/cyto/myc/GFP* and pEJ2-GFP substrates

G1-enriched H1299/ pCMV/cyto/myc/GFP* cells were infected with Ad-I-SceI-NG. Twenty-four hours later cells were subjected to flow cytometric analysis. In addition, intrachromosomal MMEJ was measured using U2OS cell with pEJ2-GFP (50). G1-enriched cells were infected with Ad-I-Sce I-NG, followed by fluorescence-activated cell sorting (FACS) analysis for green fluorescent protein (GFP) expressing cells 24 h later. For extrachromosomal MMEJ assay, G1-enriched H1299 cells were transfected with I-SceI-linearized pCMV/cyto/myc/GFP* for 4 h, followed by culture in serum-free medium for 24 h. End-joining activity was obtained by comparing GFP expression in cells transfected with linearized DNA relative to cells transfected with uncut DNA and normalized for transfection efficiency and expression levels.

### Assay of measuring MMEJ and end-resection/deletion using pGL3-MCS

The assay for NHEJ using circularization of linear plasmid substrate pGL3-MCS has been described (48). For physical analysis of repair products, plasmid DNA was extracted 24 h after transfection. DNA treated with alkaline phosphatase transformed into chemically competent cells (Invitrogen). Colonies were selected by ampicillin resistance. Mini-prep DNA plasmids were extracted from bacterial colonies (Qiagen, Hilden, Germany) and subjected to polymerase chain reaction (PCR) amplification, restriction fragment analysis and sequencing.

### Chromosomal NHEJ assay with pPHW1 reporter

To create DSB within the chromosomally integrated substrate, I-SceI endonuclease was expressed in G1-enriched H1299/pPHW1 cells by infection with Ad-SceI-NG (48,70). Genomic DNA was extracted from cells using DNeasy blood and tissue kit (Qiagen). Repair products were subjected to PCR using forward primer pPHW1-PF4 5′TCTCCGCCCCATGGCTGACTA3′; and reverse primer pPHW1-PR4 5′GTGCATGGATCTGCAACAT3′. PCR products were sub-cloned into a pMD20-T vector (Clontech) for sequencing. The sequencing primer was M13-F1- 5′ACAGGAAACAGCTATGACCA3′.

### Immunofluorescence analysis

Immunofluorescence assays were performed as published (51,52). Mouse anti-γ-H2AX (Ser139, clone JBW301, Millipore) was used at 1:500 dilution. The monoclonal anti-BrdU antibody (BD Biosciences) was used at 1:200 concentration. The secondary antibody, goat–anti-mouse immunoglobulin G (IgG) Alexa fluor 594 or fluorescein isothiocyanate-conjugated anti-mouse secondary antibody (sigma) was used at 1:500 dilution. The slides were viewed at 1000× magnification on an NIKON 90i fluorescence microscope (photometric cooled mono CCD camera).

### Cell-cycle analysis

Cells were collected and fixed with cold 70% ethanol. Approximately 10^6^/ml cells were incubated for 30 min with staining solution containing RNase A (10 μg/ml, Sigma), propidium iodide (20 μg/ml, Sigma) for 30 min. DNA content was measured by flow cytometry.

### Comet assay

M059J cells were G1 enriched by culture in medium containing 0.5 mM L-mimosine (Sigma) for 24 h. G1-enriched M059J cells were analyzed by the Comet assay under neutral conditions (Trevigen, Gaithersburg, MD, USA) as described (53). Comets were analyzed using CometScore software (TriTek, Sumerduck, VA, USA). Radiation was delivered to cultured cells using a cesium-137 gamma ray at a dose rate of 3.1 Gray /min.

### Real-time quantitative reverse transcription-PCR (qRT-PCR)

Total RNA was isolated using the RNeasy Kit (Qiagen). DNA PKcs forward primer: CTGTGCAACTTCACTAAGTCCA. DNA PKcs reverse primer CAATCTGAGGACGAATTGCCT.

Ku70 forward primer: GCTAGAAGACCTGTTGCGGAA. Ku70 reverse primer TGTTGAGCTTCAGCTTTAACCTG. LIG3 forward primer: TCACTGGCGTGATGTAAGACA.

LIG3 reverse primer: CCTGGAATGATAGAACAGGCTTT. XRCC1 forward primer: TCAAGGCAGACACTTACCGAA. XRCC1 reverse primer: TCCAACTGTAGGACCACAGAG. Experiments were carried out in triplicate for each data point. Reactions were performed using SYBR Green mix and MyiQ real-time PCR detection system (Bio-Rad). Relative mRNA levels were calculated using the comparative Ct method (Δ*Ct*).

### Immunoblotting

Cell extracts were prepared and proteins were resolved by sodium dodecyl sulphate-polyacrylamide gel electrophoresis as described previously (47,54). Rabbit anti-53BP1 were purchased from Novus and used at 1:1000 dilution. Mouse anti-BRCA1 (D-9, Santa Cruz) was used at 1:100 for western blotting. HA.11 clone 16B12 from Covance was used at 1:1000 for western blotting. Mre11 was used at 1:1000 for western blotting (Novus Biologicals). Secondary antibodies used were goat-anti-mouse IgG–horseradish peroxidase (HRP) conjugated, goat-anti-rabbit IgG–HRP conjugated at 1:2000 dilutions.

### Fluorescence *in situ* hybridization (FISH) for chromosome aberration analysis

FISH was performed using pan-telomeric peptide nucleic acid probes (47,55). The cells were irradiated (2Gy) and processed with FISH analysis 15 h after IR as published (55).

## RESULTS

### 53BP1 is required for repairing IR-induced DSBs in the G1 phase of the cell cycle

**Figure 1.**
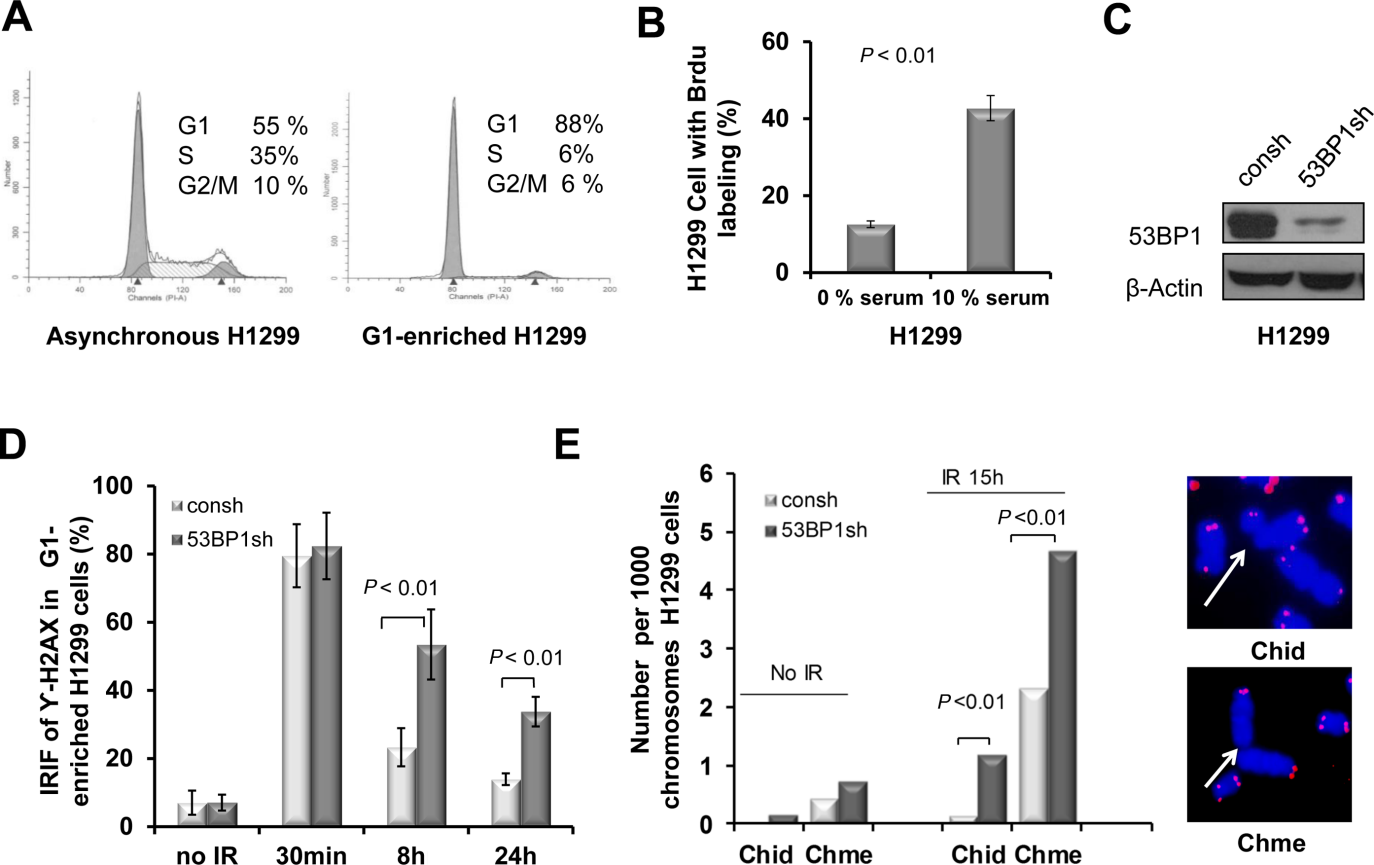
53BP1 promotes DNA DSB repair and genomic stability in G1-phase enriched cells. (**A**) Cell-cycle distribution of H1299 cells following incubation in serum-free medium for 48 h. Number of cells is plotted against DNA content determined by PI staining. (**B**) Percentage of cells with BrdU labeling in cells with or without starvation. (**C**) Transfection of H1299 cells with shRNA against 53BP1 (53BP1sh) or a control (consh). β-Actin antibody was used as a protein loading control. (**D**) Percentage of cells with ≥10 γ-H2AX foci in G1-enriched H1299 cells with or without 53BP1 depletion. *P*-values were calculated with the Student's *t*-test. Error bars represent the SD of three independent experiments. (**E**) 53BP1 depletion increases the frequency of both chromosome-type (Chme) and chromatid-type (Chid) breaks in cells collected at 15 h after 2 Gy IR. Histogram showing the frequencies of chromosome- and chromatid-type breaks (left panel). Exponentially growing H1299 cells with or without 53BP1 depletion were irradiated with 2 Gy (IR) and harvested after 15 h. At least 50 metaphases for each sample were analyzed using FISH, and at least 2000 chromosomes were counted. *P*-values were calculated with the *t*-test. Representative breaks are indicated by arrowheads (right panel). FISH using telomeric probe is indicated in pink color.

We set out to study the role of 53BP1 in DSB repair in the G1-phase of the cell cycle. H1299 cells were serum-starved for 48 h, which led to an enrichment of cells in the G1-phase and a pronounced reduction in S-phase cells (Figure 1A and B). Studies have indicated that there is a correlation between the number of foci of phosphorylated H2AX (γ-H2AX) and the number of DSBs induced by IR and that the rate of disappearance of foci reflects DSB repair, especially in G1-arrested cells (56). Thus, we next determined the effect of 53BP1 depletion on IR-induced γ-H2AX foci. 53BP1 was depleted by RNA interference (Figure 1C). Depletion of 53BP1 resulted in a statistically significant increase in the percentage of G1-enriched H1299 cells with residual IR-induced γ-H2AX foci at both 8 and 24 h time points (Figure 1D). To determine the consequences of 53BP1 depletion at the chromosomal level, we examined metaphase spreads of cells using FISH. In this assay, cells with IR-induced DSB in G1-phase can traverse S-phase and progress to the first mitosis (57). Cell collection is done at different times after irradiation, which will yield breaks originating from different cell-cycle phases (58). Here, exponentially growing cells with or without 53BP1 knockdown were irradiated and harvested after 15 h, which produced mainly cells that had been in G1-phase at the time of IR exposure. We observed a significant increase in the frequency of chromosome-type breaks, in addition to chromatid-type breaks (Figure 1E, left panel). Representative IR-induced chromosome- and chromatid-type breaks in 53BP1-depleted cells are shown in Figure 1E (right panel). Together, Figure 1 suggests that 53BP1 is important for the repair of DSBs in G1 phase cells.

### 53BP1 promotes DSB repair independently of DNA-PKcs

**Figure 2.**
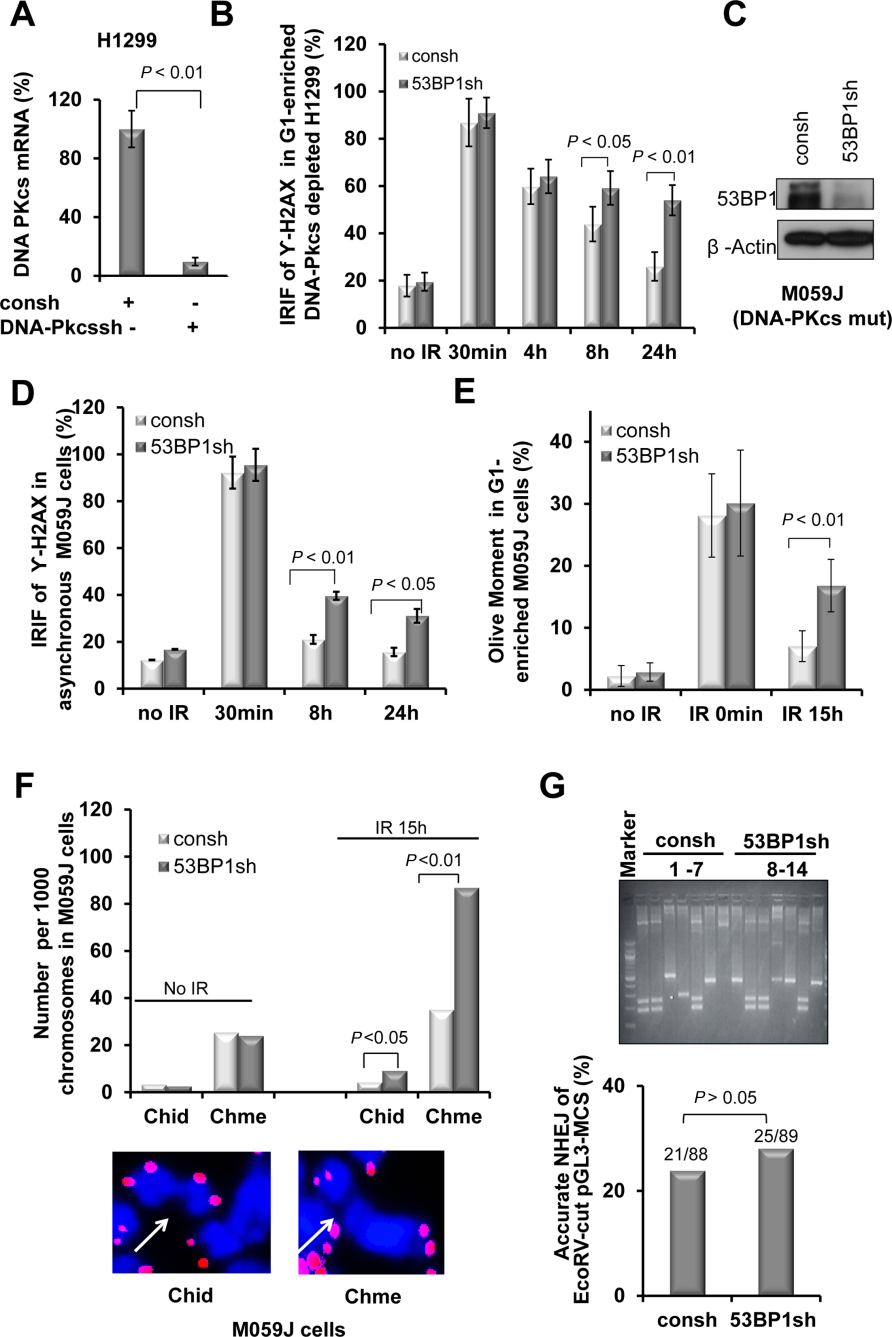
53BP1 promotes DNA DSB repair and genomic stability in cells with deficient DNA-PKcs. (**A**) Relative DNA-PKcs mRNA levels from H1299 cells transfected with shRNA against DNA-PKcs (DNA-PKcssh) or a control (consh). (**B**) Percentage of cells with ≥10 γ**-**H2AX foci with or without 53BP1 depletion in irradiated G1-enriched H1299 cells with additional DNA-PKcs depletion. (**C**) Western blot showing 53BP1 depletion in M059J cells. (**D**) Percentage of cells with γ-H2AX foci in irradiated asychronous M059J cells. (**E**) Olive moment in the Comet assay with or without 53BP1 depletion in G1-enriched M059J cells. *P*-values were calculated with the Student's *t*-test. Error bars represent the SD of three independent experiments. (**F**) 53BP1 depletion increases the frequency of both Chme and Chid breaks in M059J cells harvested at 15 h after IR (upper panel). The methods are the same as described in Figure 1E. (**G**) A representative spectrum of individual repair products recovered from G1-enriched H1299 cells with or without 53BP1 transfected with EcoRV-linearized pGL3-MCS substrate (59) (upper panel). The frequency of precise NHEJ was determined by DNA sequencing (bottom panel). *P-*values were calculated by Chi-square test.

The functional interaction between 53BP1 and DNA-PKcs has been incompletely studied (8). To address if 53BP1 promotes DSBs repair via DNA-Pkcs in our system, we first knocked down DNA-PKcs in H1299 cells (Figure 2A). Additional depletion of 53BP1 led to an increase in the fraction of cells with residual γ-H2AX foci (Figure 2B), similar to the effect seen in DNA-PKcs-proficient cells (Figure 1D). We confirmed the independence of 53BP1 from DNA-PKcs function by knocking down 53BP1 in M059J cells which lack the catalytic subunit of DNA-PKcs (Figure 2C). An increase in residual DSB upon 53BP1 depletion using γ-H2AX foci and Comet assays was seen in both asynchronous (Figure 2D) and G1-enriched cell populations (Figure 2E, Supplementary Figure S1). Next, we determined chromosome aberrations in M059J cells. Similar to the results described in Figure 1E, 53BP1 knockdown in these cells led to a significant increase in chromosome-type breaks as well as chromatid-type breaks (Figure 2F), indicating that 53BP1 maintains genomic stability independent of DNA-PKcs in G1 phase cells.

To further confirm that the function of 53BP1 is independent of DNA-PK, we assessed the impact of 53BP1 depletion on accurate NHEJ in an extrachromosomal reporter substrate, pGL3-MCS, according to our previous publication (59) (Supplementary Figure S2A). After transfection of the *EcoRV*-linearized substrate in G1-enriched cells, plasmids were extracted and analyzed after 24 h. Substrates undergoing accurate rejoining retained the *Eco*RV restriction site as determined by PCR with primers flanking the break site. Representative NHEJ products derived from cells with or without 53BP1 shRNA transfection are displayed in Figure 2G, upper panel. Quantification of products showed similar frequencies of accurate NHEJ with or without functional 53BP1 (Figure 2G, lower panel). Similar results were obtained with a second reporter, pCMV/myc/cyto/GFP, in the asychronized and G1-enriched cells (Supplementary Figure S2B–D). This reporter was assayed extrachromosmally as a *BmtI*-linearized substrate, and was previously utilized to similarly detect accurate NHEJ (Supplementary Figure S2B) (30). Altogether, from the data in Figure 2, we conclude that 53BP1 promotes DSB repair in G1-phase cells independently of DNA-PKcs.

### 53BP1 promotes MMEJ in G1-enriched cells

**Figure 3.**
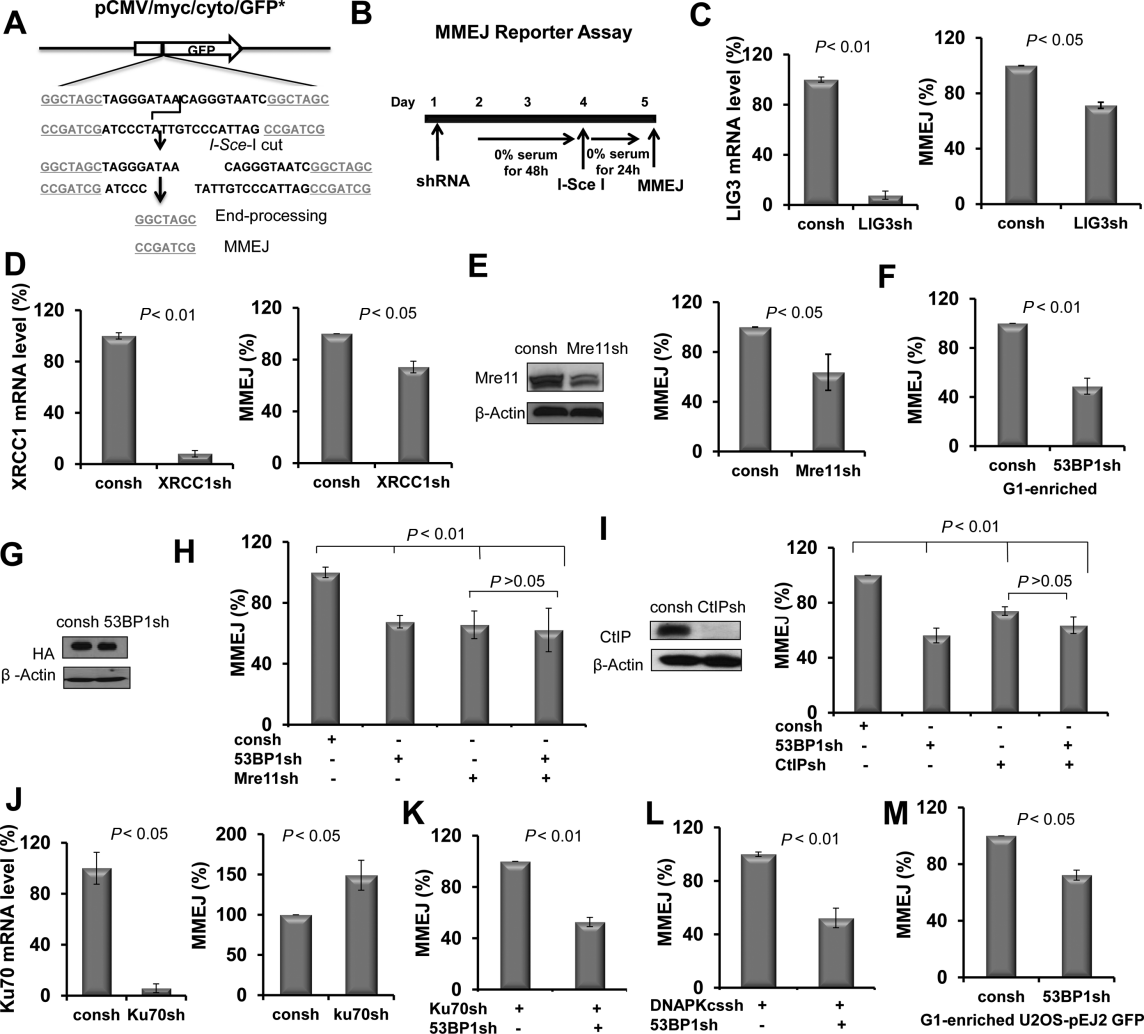
53BP1 knockdown led to a reduction in MMEJ in G1-enriched cells. (**A**) Schematic maps of reporter plasmid. The vector pCMV/myc/cyto/GFP* was modified by inserting a 28-bp oligonucleotide containing an I-SceI site flanked by 7 bp microhomology (underlined) inside the GFP sequence. This insertion introduces two stop codons and a frameshift, preventing GFP translation. Functional GFP is only generated after I-SceI cutting and subsequent repair by MMEJ. (**B**) Illustration of the chromosomal MMEJ assay. (**C–E**) The effect of knockdown of the individual proteins as indicated on MMEJ in G1-enriched H1299 cells. (**F**) 53BP1 knockdown led to a reduction in MMEJ frequency in G1-enriched cells. (**G**) Expression of HA-tagged I-SceI endonuclease was monitored by western blot using anti-HA antibody. H1299-pCMV/myc/cyto/GFP* cells with and without 53BP1 knockdown were infected with Ad-I-SceI-NG, and whole cell lysates were prepared at 24 h after infection. (**H**) 53BP1 promotes MMEJ in a manner that depends on Mre11. (**I**) CtIP was knocked down by shRNA targeting CtIP (CtIPsh) (left panel). The role of 53BP1 in MMEJ depends on CtIP (right panel). (**J**) Ku70 depletion by shRNA (Ku70sh) in H1299 cells. (**K**) The effect of knockdown of 53BP1 on MMEJ frequency in G1-enriched H1299 cells with additional Ku depletion. (**L**) The effect of knockdown of 53BP1 on MMEJ frequency in G1-enriched H1299 cells with additional DNA-pkcs depletion. (**M**) 53BP1 depletion results in a decreased frequency of MMEJ in G1-enriched U2OS-EJ2-GFP cells. All *P-*values were calculated by Student's *t-*test. Error bars represent the SD of at least three independent experiments.

Because the repair of IR-induced DSBs predominately depends on Alt-NHEJ in DNA-PKcs-deficient M059J cells (9,10), we hypothesized that 53BP1 promotes Alt-NHEJ. Although it is not clear whether microhomology is absolutely required for Alt-NHEJ, microhomology sequences are frequenctly observed at the junction sites. Therefore, we wished to specifically examine the role of 53BP1 in MMEJ, which is dependent on Mre11 and CtIP among other factors (8,16,18,29–32). We, therefore, established H1299 clones with a single chromosomally integrated copy of the pCMV/myc/cyto/GFP* reporter. The reporter is modified by inserting a 28-bp oligonucleotide containing an I-SceI recognition site flanked by 7-bp microhomology into the GFP sequence as described previously (30,48). This insertion introduces two stop codons and a frame shift preventing GFP translation. Functional GFP is only generated after I-SceI cleavage and subsequent repair by MMEJ with 26 bp depletion (Figure 3A). This reporter was previously used to describe the role of CtIP in MMEJ G1-phase cells (30). The protocol for MMEJ measurement in G1-enriched cells is presented in Figure 3B. In this assay, shRNA-transfected cells are first enriched in G1 phase by starvation followed by infection with an adenoviral I-SceI expression vector and FACS analysis of GFP-expressing cells. MMEJ was detected 24 hours after I-SceI is introduced into the cells. Brdu labeling is also monitored right prior to MMEJ detection in order to ensure that G1 enrichment is efficient throughout the experiment (Supplementary Figure S3).

Depletion of DNA ligase III and XRCC1 as well as Mre11 led to an expected decrease in the percentage of MMEJ products, confirming that the reporter is an appropriate endpoint to study the involvement of 53BP1 in this pathway (Figure 3C–E). The observed smaller magnitude of effect in ligase III and XRCC1 depleted cells may be caused by functional redundancy as has been reported for ligase I and ligase III in alt-NHEJ (60,61). We found depletion of 53BP1 led to a decrease in MMEJ frequency that was of similar magnitude as Mre11 depletion (Figure 3F) and seen extrachromosomally as well (Supplementary Figure S4A). The expression of I-SceI was monitored by western blot using antibody against the HA tag in cells with or without 53BP1 knockdown. shRNA transfection did not affect expression of I-SceI protein levels (Figure 3G).

The role of 53BP1 in promoting MMEJ appeared to be G1-phase specific and was not observed in asynchronous cells (Supplementary Figure S4B). The effect of 53BP1 appear to be epistatic with Mre11 (Figure 3H) as well as CtIP (Figure 3I), thereby demonstrating that 53BP1 acts in the genetically defined MMEJ pathway. Importantly, knockdown of the c-NHEJ protein Ku70 resulted in increased MMEJ levels, which is consistent with previous observations that the MMEJ pathway is suppressed by Ku (18) (Figure 3J). In the setting of Ku70 knockdown, additional depletion of 53BP1 again resulted in a decrease in MMEJ frequency in G1-enriched cells (Figure 3K). Importantly, we also found that depletion of 53BP1 reduced the frequency of MMEJ in G1-enriched cells with a DNA-PKcs deficiency (Figure 3L). This result is consistent with the data in Figure 2 which demonstrates a DNA-PKcs-independent DSB repair function of 53BP1. Finally, in order to compare our data with others, we utilized a second chromatinized MMEJ reporter, pEJ2-GFP, which has been used to study the role of 53BP1 in Alt-NHEJ in asynchronous cells (50) (Supplementary Figure S5A). In this system, a 35 or 140–350 bp deletion is required during the MMEJ process (18,50). Similar to the results observed in H1299 cells with the pCMV/myc/cyto/GFP* reporter (Figure 3F), 53BP1 knockdown impaired MMEJ in G1-enriched U2OS cells harboring the pEJ2-GFP reporter (Figure 3M, Supplementary Figure S5B). In addition, consistent with a previous publication (50), 53BP1 knockdown caused a slight increase in MMEJ in asynchronous cells using the same reporter (Supplementary Figure S5C). Altogether, the data in Figure 3 indicates that 53BP1 promotes MMEJ specifically in G1 phase.

### 53BP1 specifically promotes MMEJ with extensive resection/deletion

**Figure 4.**
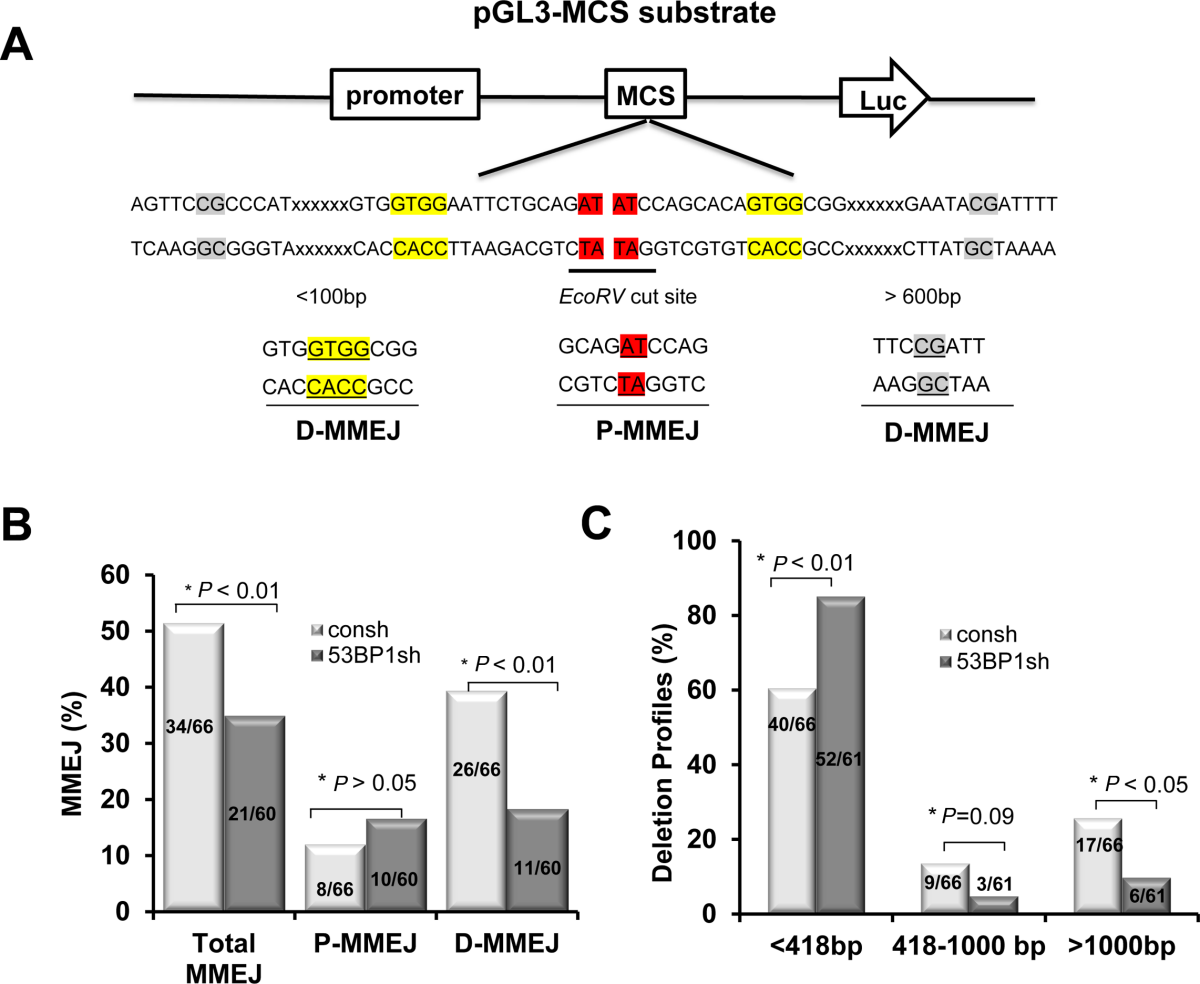
53BP1 is required for distal MMEJ with extensive end resection/deletion. (**A**) Schema of reporter substrate and illustration of microhomology usage. Representative microhomologies used during proximal MMEJ (p-MMEJ) and distal MMEJ (d-MMEJ) were marked in red and yellow/gray colors, respectively. (**B**) Levels of MMEJ determined by sequencing analysis of individual re-circularized pGL3-MCS products. P-MMEJ represents repair adjacent to the *EcoRV* cleavage site. D-MMEJ represents repair distant to the *EcoRV* site with extensive end resection/deletion. (**C**) Quantitative analysis of deletion products by sequence analysis of individual re-circularized pGL3-MCS products. *P-*values were calculated by Chi-square test.

The pGL3-MCS reporter allows us to assay not only accurate NHEJ events by analyzing repair products that have retained the EcoRV-site but also MMEJ and the size of sequence deletion around the cleavage site (Supplementary Figure S2A, Figure 2G). To further analyze the effect of 53BP1 on sequence deletion during the MMEJ process, we sequenced repair products of pGL3-MCS that could not be cleaved by *EcoRV* endonuclease. The reporter allows for two types of end-joining, proximal MMEJ (P-MMEJ), which microhomology sequence is flanking the DBS site and involves sequence deletion of only 2 bp prior to re-ligation, and distal MMEJ (D-MMEJ), which can be mediated by microhomologies that are located 4–2364 bp away from the break site yielding 26 different repair products (Figure 4A and B). In cells with 53BP1 knockdown, there was a significant decrease in the fraction of products representing distal MMEJ, but not proximal MMEJ (Figure 4B). Since a length of microhomology that would clearly distinguish c-NHEJ and alt-NHEJ is not defined, we acknowledge that P-MMEJ may also be carried out by c-NHEJ. However, even if this were the case, it would no alter our conclusion that 53BP1 promotes MMEJ (Figure 4B). We next categorized the deletions into three arbitrary classes, i.e. ≤418 bp versus 418–1000 bp versus >1000 bp. In the presence of 53BP1, almost 60% of deletions were ≤418 bp, whereas in 25% of products the deletion size exceeded 1000 bp (Figure 4C). Conversely, 53BP1 depletion was associated with a higher percentage of ≤418 bp deletions (85%) and a lower percentage of large deletions (10%). In summary, the data indicate that the promotion of MMEJ by 53BP1 is associated with extended deletions and thus is error-prone, in contrast to c-NHEJ.

### Loss of MMEJ promotion by 53BP1 when BRCA1 is defective

**Figure 5.**
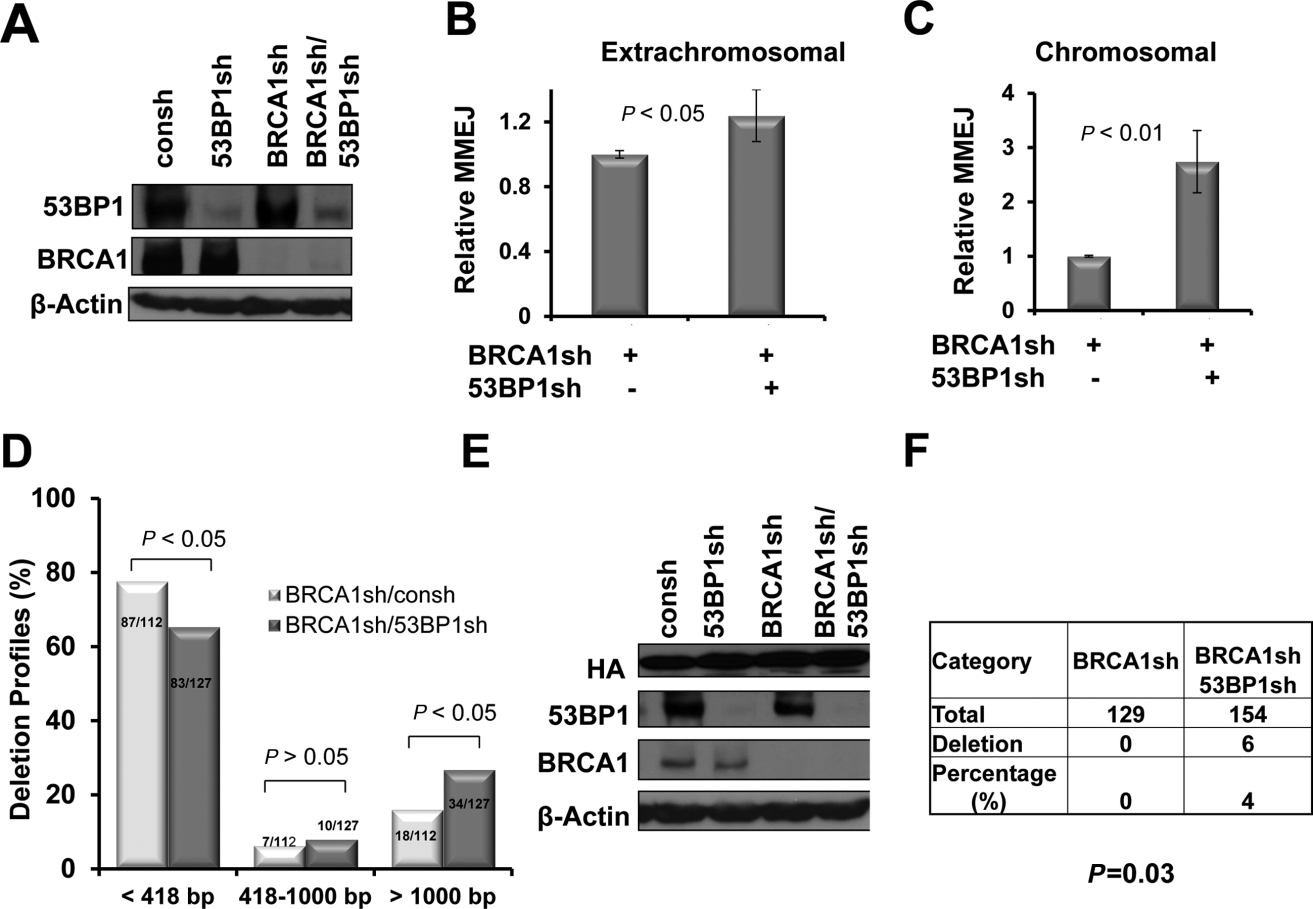
53BP1 inhibits MMEJ and end-resection/deletion in the BRCA1-deficient cells. (**A**) Protein levels of 53BP1 in H1299 cells with or without BRCA1 depletion were detected by western blot using an anti-BRCA1/53BP1 antibody or β-actin as a protein loading control. (**B** and **C**) MMEJ measured by the extrachromosomal or chromatinized reporter pCMV/myc/cyto/GFP* in G1-enriched cells, according to the protocol described in Figure 3A, *P-*values were calculated by Student's *t-*test. Error bars represent the SD of three independent experiments. (**D**) Quantitative analysis of deletion products by sequence analysis of individual re-circularized pGL3-MCS in cells depleted of the indicated genes. (**E**) HA-I-SceI expression and BRCA1/53BP1 expression are monitored by western blot. (**F**) Quantitation of changes in NHEJ events. A cell clone with a single copy of pPHW1 (H1299-pPHW1) was enriched in G1 phase using starvation as described in Figure 3A. To avoid selection bias, colonies did not undergo XHATM selection for gpt expression. *P* < 0.05, *P-*values were calculated by Chi-square test.

BRCA1 and 53BP1 have opposing functions in the regulation of HR. We thus wished to examine whether 53BP1's role in MMEJ promotion is affected by BRCA1 status. We used the pCMV/myc/cyto/GFP* reporter according to the protocol described in Figure 3B. 53BP1 depletion did not result in reduced but rather increased MMEJ frequencies in G1-enriched populations in which BRCA1 was depleted compared to control cells (Figure 5A–C), contrasting the results of 53BP1 depletion in the context of proficient BRCA1 (Figures 3 and 4B). We next analyzed the deletion profile of repair products using the extrachromosomal pGL3-MCS reporter analogously to Figure 4. Interestingly, the proportion of the junction products with a larger deletion (>1000) was higher in cells with double knockdown of 53BP1 and BRCA1 compared to cells with BRCA1 depletion alone (Figure 5D), contrasting the results of 53BP1 depletion in a BRCA1-proficient background (Figure 4C).

To confirm these findings at a chromosomal level, we generated H1299 cells in which a single copy of the I-SceI-based NHEJ substrate pPHW1 was stably chromosomally integrated (see Materials and Methods). This repair substrate was described previously (62,63) and is depicted in Supplementary Figure S6. Following I-SceI break induction, we analyzed profiles of the repair junctions by direct sequencing of unselected products from G1-enriched populations. Equivalent expressions of HA-tagged I-SceI meganuclease and BRCA1/53BP1 knockdown were monitored in parallel by western blotting (Figure 5E). Without 53BP1 silencing, we could not detect any deletion among 129 clones in the presence of BRCA1 depletion (Figure 5F). In contrast, double depletion of BRCA1 and 53BP1 revealed 6 deletions out of 154 clones, with a deletion size of 4–26 bp. Therefore, BRCA1 modulates 53BP1's role in regulating deletional MMEJ in G1-phase cells.

## DISCUSSION

### 53BP1 promotes MMEJ in G1-phase cells

In this report, we have demonstrated a novel role of 53BP1 in promoting MMEJ and sequence resection/deletion in G1-phase cells. Our results provide a mechanistic basis for prior observations showing that 53BP1 is important for cell survival following irradiation of G1-phase cells in a Ku-independent manner (34,35). A previous report suggested that 53BP1 may promote DSB repair in late G1-phase via promotion of heterochromatin relaxation (41). Our resutly imply a role of 53BP1 in MMEJ. In addition, we found that 53BP1 promotes deletion of sequence flanking broken ends, raising the possibility that 53BP1 promotes DNA end-resection during MMEJ. 53BP1 contains interaction surfaces for numerous DSB-responsive proteins although it does not possess any identified nuclease enzymatic activities. Thus, the effect of 53BP1 on ssDNA resection could be indirect via association with its partner(s) which play a direct role in ssDNA resection. Given that 53BP1 directly interacts with and regulates the activity and recruitment of Mre11 (41,64), it is conceivable that 53BP1 promotes end-resection/degradation via regulation of Mre11. In support of this hypothesis, using shRNA knockdown approach we found that promotion of MMEJ by 53BP1 was dependent on Mre11 and CtIP which promotes ssDNA resection via Mre11 (Figure 3H and I) (64,65).

Previously, a role of 53BP1 in inhibition of end-resection/degradation in class switch recombination (CSR) has been observed (66). In addition, 53BP1 was found to promote end-resection/degradation during long-range V(D)J recombination (38). The authors proposed a model in which the long-range synapsis mediated by 53BP1 is critical for protecting DNA ends during V(D)J recombination and CSR (38). Currently, it is not clear if 53BP1 acts on synaptic steps during repair of DSBs induced by IR or other exogenous sources. It is possible that 53BP1 has a similar role in long-range synapsis of DSBs caused by IR or other sources. On one hand, 53BP1 could hold the long ranged DSB ends together directly or indirectly to protect extensive degradation during synapsis. On the other hand, the internal regions can be subsequently resected/degraded via 53BP1-mediated regulation of Mre11/CtIP. In this scenario, this could allow for controlled end-resection induced by Mre11/CtIP. Although 53BP1 may promotes end-resection/degradation during MMEJ, the magnitude of sequence deletion may be limited because ssDNA resection activity is generally low in G1 phase due to the lower CDK activity and/or the restriction by H2AX (67).

### 53BP1 is required for maintenance of genomic stability in G1-phase cells with a deficiency in DNA-PK

We found that 53BP1 depletion caused an increase in chromosome-type breaks in G1-phase cells with or without DNA-Pkcs deficiency (Figures 1E and 2F). These results suggest that 53BP1 is required for maintenance of genomic stability via promotion of DSB repair in G1-phase cells with a deficiency in the c-NHEJ pathway. In addition, we observed that IR-induced chromatid-type breaks were also more common in 53BP1-depleted cells. We speculate that this observation may reflect a role of 53BP1 in the repair of secondary DSBs that are generated after replication blockage caused by IR though we did not pursue this further (68). The role of 53BP1 in MMEJ may be important for the genomic stability of G1-phase cells due to the following reasons. First, no role of 53BP1 in precise error-free end-joining, which reflects C-NHEJ was observed. Second, 53BP1 was found to be important for repairing of DSBs in DNA-PKcs-deficient G1 cells (Figure 2). Thus, it is tempting to speculate that the role of 53BP1 in alt-NHEJ/MMEJ is required for repair of DSBs in G1 phase cells in response to IR. We note that our data do not rule out the possibility that some DSBs may be repaired by 53BP1 in concert with DNA-PKcs, particularly complex lesions that require some degree of end-processing.

### Upregulation of MMEJ with deletion formation when both 53BP1 and BRCA1 functions are impaired

Interestingly, the MMEJ-promoting role of 53BP1 in G1-phase cells was lost when BRCA1 was depleted (Figure 5). We speculate that this could reflect an active suppression of end-resection by 53BP1 in the absence of BRCA1. Such a scenario would mirror 53BP1's inhibitory role in ssDNA resection that is required for HR (42,43). MMEJ shares the initial resection step with HR (13) but it differs from HR because it requires neither extended resection nor extended sequence homology. The potential mechanisms by which 53BP1 may actively suppress ssDNA deletion/resection required for MMEJ in G1-phase cells with BRCA1 deficiency are far from clear. Several studies have suggested that the blockage of DNA-end-resection required for HR in BRCA1-deficient cells involves preventing the phosphorylation of RPA and/or the action of ATM-dependent CtIP (42,43). In addition, 53BP1 blocks 5′-end-resection that involves CtIP, BLM and Exo1 at telomeres deprived of both TRF1 and TRF2 (69). Further, 53BP1 counteracts BRCA1 in suppressing ssDNA resection via association with RIF1 (70–73). It would be very interesting to determine if the mechanisms inhibiting ssDNA resection by 53BP1 during HR repair, telomeres maintenance and class switch reocmbination/long-ranged V(D)J recombination are also utilized during the process of MMEJ. In addition, the question of how 53BP1 acts differently in MMEJ in presence and absence of BRCA1 need to be addressed in future.

One remaining question is how 53BP1 acts differently in ssDNA resection and DSB repair in cells with or without BRCA1. In BRCA1-proficient S-phase cells, 53BP1 has no role in ssDNA resection required for HR (45), but 53BP1 has an inhibitory impact on resection/HR in BRCA1-deficient cells (42,43). In G2-phase cells, there is a HR-promoting role of 53BP1, which may be a reflection of relaxation of heterochromatin. Nonetheless, 53BP1 still retains its inhibitory impact on HR. Thus, in G2-phase cells, 53BP1 has both an inhibitory and a promoting role for HR (45). We hypothesize that a similar scenario may also exist for the MMEJ pathway in G1-phase cells where 53BP1 may be both a negative and positive regulator of ssDNA resection and MMEJ. However, the suppression of ssDNA resection by 53BP1 in G1-phase cells is relieved upon loss of BRCA1 which exceeds the positive regulation by 53BP1. Thus, the net effect of 53BP1 on MMEJ in G1-phase cells is inhibitory when BRCA1 is lost. This model needs to be tested in future studies. Together, the current literature and our results strongly suggest that 53BP1's role in DSB repair is highly regulated, and depends on the stages of cell cycle and state of BRCA1.

Notably, despite the hypersensitivity to IR caused by 53BP1 loss in several, diverse cell and animal models radiation resistance was paradoxically observed in breast cancer patients with low 53BP1 expression (74). It is conceivable that cancers with low 53BP1 expression have a co-existing defect in the BRCA1 pathway given that 53BP1 loss is frequently observed in BRCA-deficient cells (42). Given that MMEJ is important for resistance to IR (14), increased MMEJ levels in BRCA1-deficient cells due to lower 53BP1 expression could contribute to radiation resistance. Thus, the possibility of resistance to IR treatment may have to be considered when breast cancer patients with a deficiency in both BRCA1 and 53BP1 are treated with IR in the clinic.

In conclusion, our data indicate that 53BP1 controls sequence deletion which may reflect ssDNA resection during MMEJ in the G1 phase. Importantly, 53BP1 appears to suppress deletion formation when BRCA1 is deficient. MMEJ, therefore, is a highly regulated DSB repair pathway and is differentially regulated in the presence or absence of BRCA1.

## SUPPLEMENTARY DATA

Supplementary Data are available at NAR Online.

SUPPLEMENTARY DATA
