# Supplementary material for: 53BP1 promotes microhomology-mediated end-joining in G1-phase cells
Source: Nucleic Acids Res. 2015 Jan 13;43(3):1659–70. doi: 10.1093/nar/gku1406 (PMC4330367; doi:10.1093/nar/gku1406)
Supplement: SUPPLEMENTARY DATA [file supp_43_3_1659__index.html]

53BP1 promotes microhomology-mediated end-joining in G1-phase cells — SUPPLEMENTARY DATA 

# 53BP1 promotes microhomology-mediated end-joining in G1-phase cells

## SUPPLEMENTARY DATA

**Files in this Data Supplement:**

- SUPPLEMENTARY DATA
