# Supplementary material for: 53BP1 promotes microhomology-mediated end-joining in G1-phase cells
Source: Nucleic Acids Res. 2015 Jan 13;43(3):1659–70. doi: 10.1093/nar/gku1406 (PMC4330367; doi:10.1093/nar/gku1406)
Supplement: SUPPLEMENTARY DATA [file supp_gku1406_nar-03151-d-2014-File007.pdf]

Figure S1

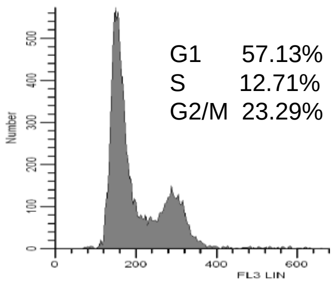

Asynchronous M059J

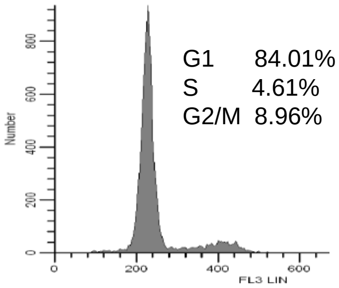

G1-enriched M059J

Figure S2

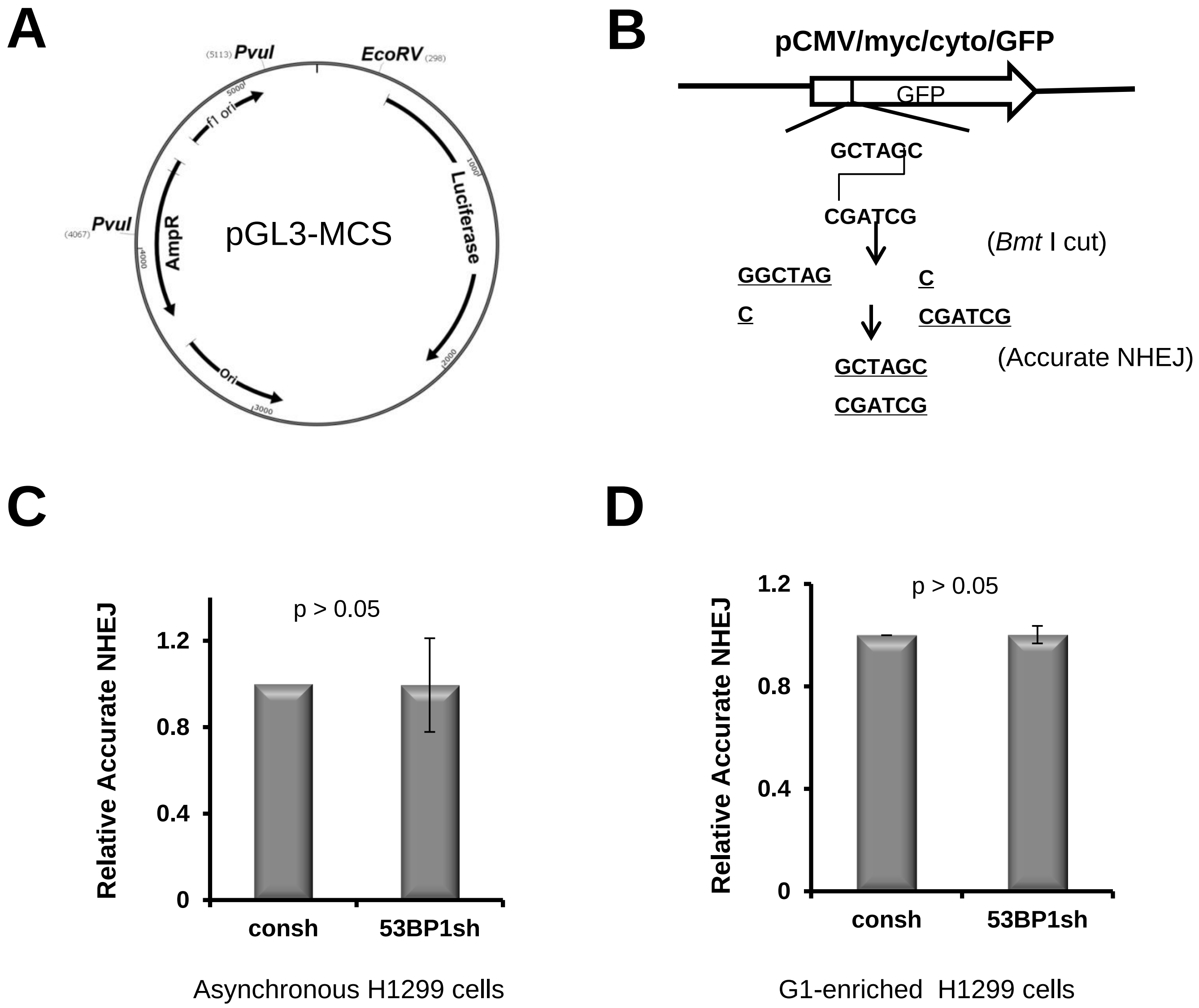
A
(5113) PvuI
EcoRV (298)
5000
f1 ori
1000
Luciferase
(4067) PvuI
4000
AmpR
pGL3-MCS
2000
Ori
3000
B
pCMV/myc/cyto/GFP
GFP
GCTAGC
CGATCG
(Bmt I cut)
GGCTAG
C
C
CGATCG
(Accurate NHEJ)
GCTAGC
CGATCG
C
Relative Accurate NHEJ
p > 0.05
1.2
0.8
0.4
0
consh
53BP1sh
Asynchronous H1299 cells
D
Relative Accurate NHEJ
1.2
p > 0.05
0.8
0.4
0
consh
53BP1sh
G1-enriched H1299 cells

Figure S3

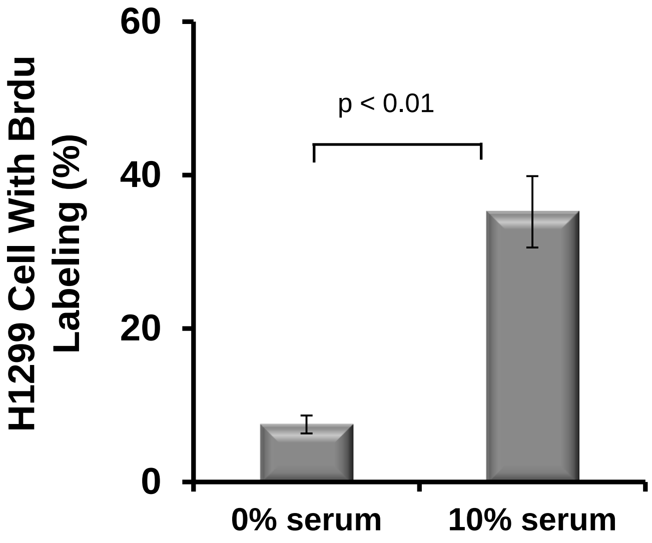

Figure S4

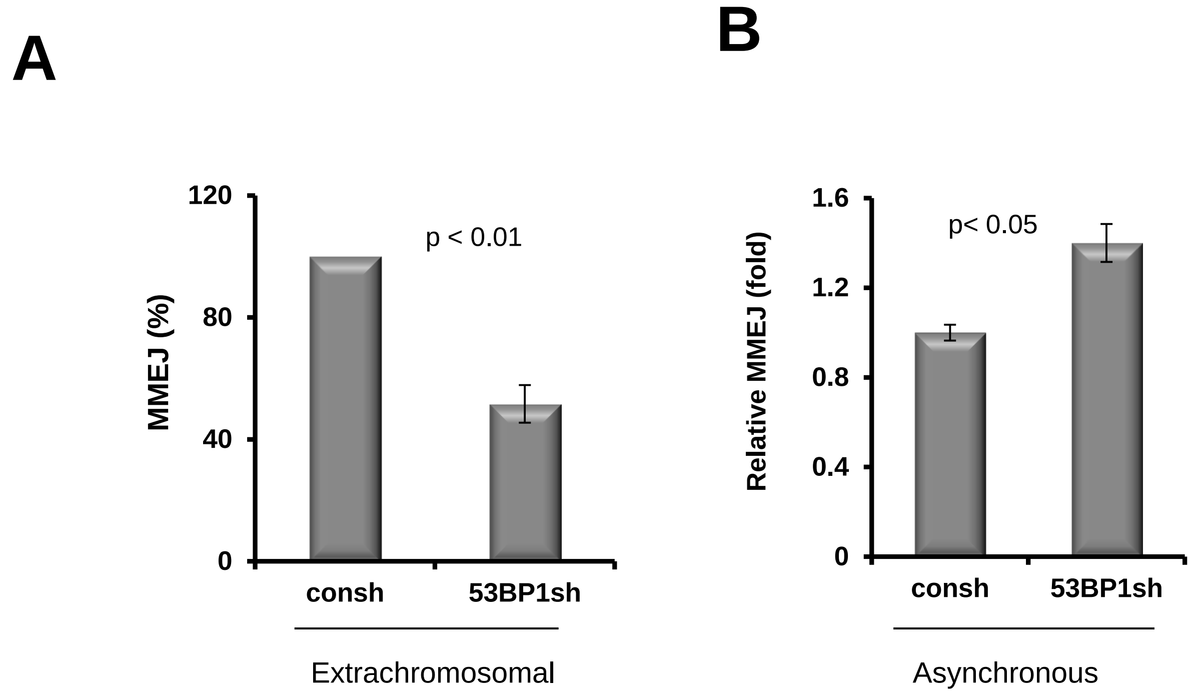
A
B
120
80
40
0
MMEJ (%)
p < 0.01
consh
53BP1sh
Extrachromosomal
1.6
1.2
0.8
0.4
0
Relative MMEJ (fold)
p< 0.05
consh
53BP1sh
Asynchronous

Figure S5

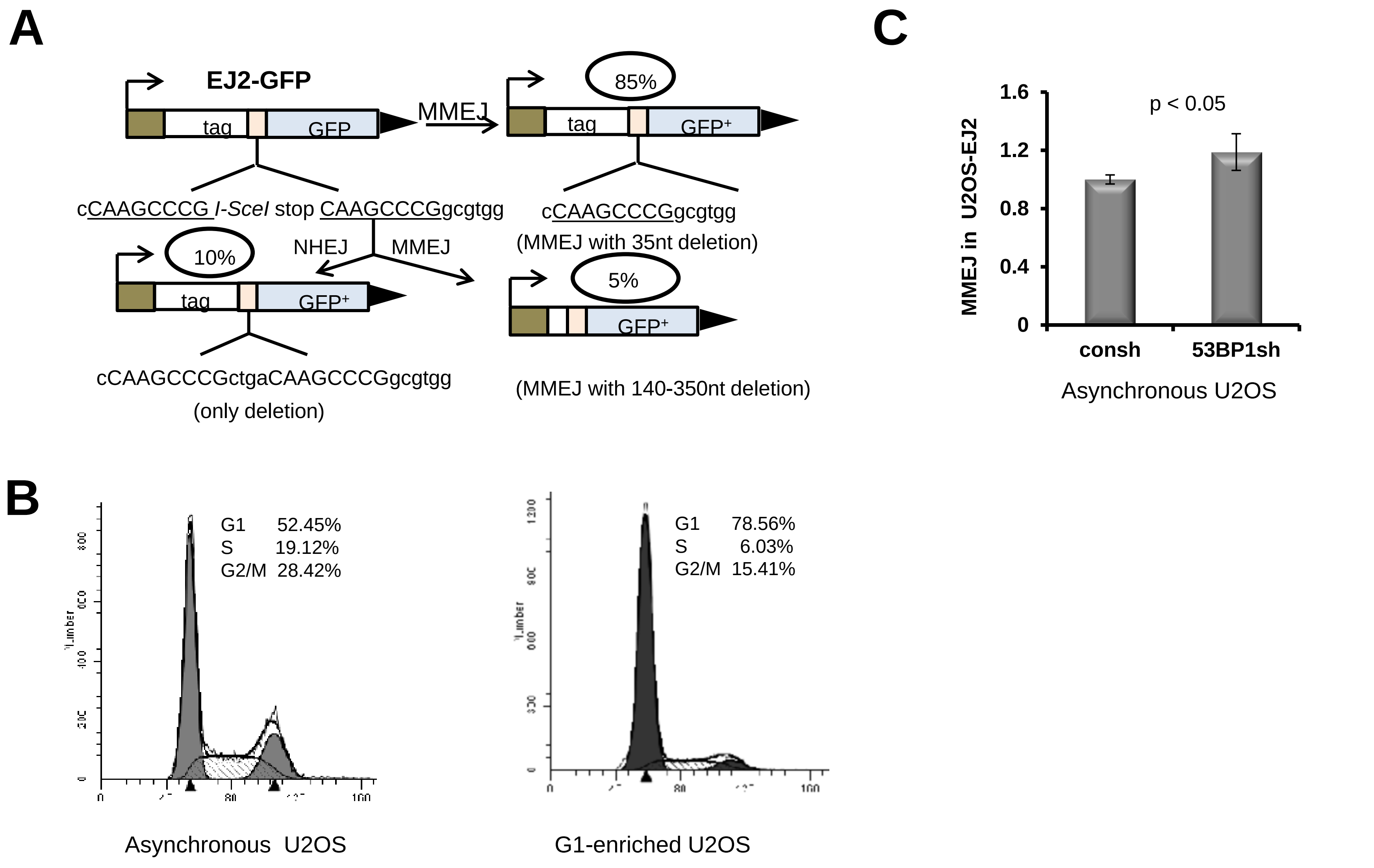
A
EJ2-GFP
tag
GFP
MMEJ
cCAAGCCCG I-SceI stop CAAGCCCGgcgtgg
85%
tag
GFP+
cCAAGCCCGgcgtgg
(MMEJ with 35nt deletion)
NHEJ
MMEJ
10%
tag
GFP+
cCAAGCCCGctgaCAAGCCCGgcgtgg
(only deletion)
5%
GFP+
(MMEJ with 140-350nt deletion)
B
G1 52.45%
S 19.12%
G2/M 28.42%
Asynchronous U2OS
G1 78.56%
S 6.03%
G2/M 15.41%
G1-enriched U2OS
C
MMEJ in U2OS-EJ2
1.6
1.2
0.8
0.4
0
p < 0.05
consh
53BP1sh
Asynchronous U2OS

Figure S6

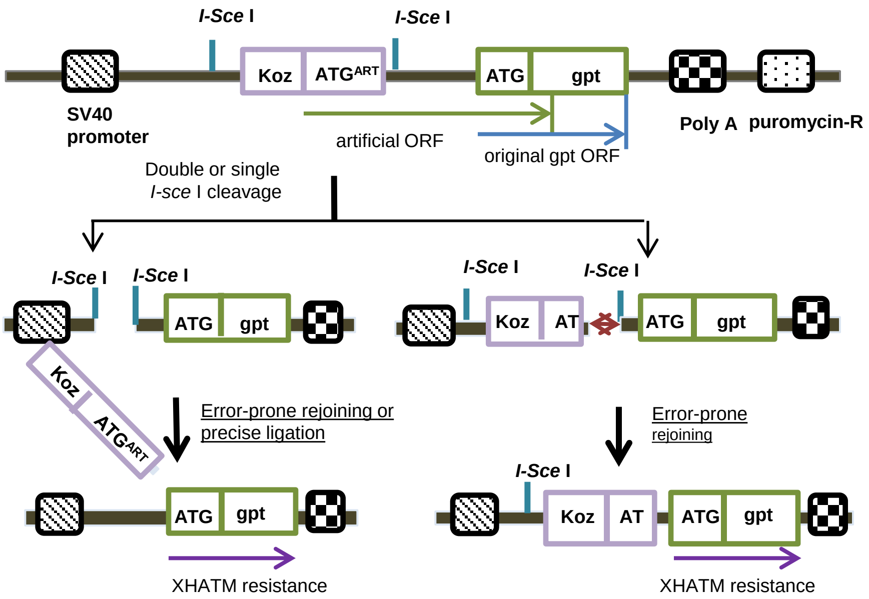

I-Sce I
I-Sce I
Koz
ATG^ART
ATG
gpt
SV40 promoter
artificial ORF
original gpt ORF
Poly A
puromycin-R
Double or single
I-sce I cleavage
I-Sce I
I-Sce I
ATG
gpt
I-Sce I
I-Sce I
Koz
AT
ATG
gpt
Koz
ATG^ART
Error-prone rejoining or precise ligation
Error-prone rejoining
ATG
gpt
XHATM resistance
I-Sce I
Koz
AT
ATG
gpt
XHATM resistance

Figure S1. Synchronization of M059J cells. Cell cycle distribution following G1 synchronization with 0.5mM L-mimosine for 24 hr in M059J cells. Number of cells is plotted against DNA content determined by PI staining.

Figure S2. 53BP1 has no role in accurate NHEJ. (**A**) pGL3-MCS offers a set of unique restriction enzyme cleavage sites, including EcoRV, between the promoter and the LUC gene. The MMEJ event can be analyzed by sequencing the repair products of linearized pGL3-MCS. (**B**) Schematic map of reporter detecting precise NHEJ. Linearized pCMV/myc/cyto/GFP with *Bmt*I generates ends with 5'-overhang and disrupts the GFP coding sequence unless it is accurately re-ligated. (**C**) 53BP1 depletion shows no significant reductions in accurate NHEJ using the reporter of pCMV/myc/cyto/GFP in asynchronized H1299 cells. (**D**) 53BP1 knockdown shows no effect in accurate NHEJ in G1-enriched H1299 cells using the pCMV/myc/cyto/GFP reporter. *P*-values were calculated by Student's *t*-test. Error bars represent the SD of three independent experiments.

Figure S3. Percentage of cells with BrdU labeling in cells with or without starvation after I-Sce-I virus are introduced into cells for 24 hr.

Figure S4. The effect of 53BP1 in MMEJ. (**A**) 53BP1 depletion results in a decreased frequency of MMEJ using an extrachromosomal MMEJ reporter, pCMV/myc/cyto/GFP* in G1-enriched cells. After transfection with I-SceI linearized MMEJ reporter, the starved cells were incubated in the serum-free medium for 24 hr, and then MMEJ was analyzed by flow cytometry. *P*-values were calculated by Student's *t*-test. Error bars represent the SD of three independent experiments. (**B**) 53BP1 knockdown led to an increased MMEJ frequency in asynchronous cells. The experiments were repeated at least four times and error bars are SD. ($p < 0.05$, *t*-test).

Figure S5. Role of 53BP1 in MMEJ in asynchronous U2OS cells with integrated pEJ2-GFP reporter. (**A**) Schematic maps of reporters. The vector EJ2-GFP is shown with three NHEJ products that are found to result in GFP^+ cells. The predominant GFP^+ product (85%) results from a 35 nt deletion and a minority GFP^+ result (5%) from 140-350bp depletion, both of which are due to MMEJ repair. (**B**) Cell cycle distribution following L-mimosine treatment for 24hr. (**C**) 53BP1 depletion leads to an increase in MMEJ in asynchronous U2OS-EJ2-GFP cells. *P*-values were calculated by Student's *t*-test. Error bars represent the SD of three independent experiments.

Figure S6. A diagram of construction of NHEJ substrate pPHW1. An artificial ORF is dominant over the downstream gpt ORF. Reconstituted translation of the gpt ORF will be resistant to XHATM selection. Both precise and deletion during NHEJ can be detected.
